# Screening of Fungi Isolated from Environmental Samples for Xylanase and Cellulase Production

**DOI:** 10.1155/2013/283423

**Published:** 2013-12-12

**Authors:** Mohammed Inuwa Ja'afaru

**Affiliations:** Department of Microbiology, Modibbo Adama University of Technology, PMB 2076, Yola 64001, Nigeria

## Abstract

The aim of this work is to select filamentous fungal strains isolated from saw dust, soil, and decaying wood with the potential to produce xylanase and cellulase enzymes. A total of 110 fungi were isolated. Fifty-seven (57) of these fungi were isolated from soil samples, 32 from sawdust, and 19 from decaying wood. *Trichoderma* and *Aspergillus* had the highest relative occurrence of 42.6% and 40.8%, respectively. *Trichoderma viride* Fd18 showed the highest specific activity of 1.30 U mg^−1^ protein for xylanase, while the highest cellulase activity of 1.23 U mg^−1^ was shown by *Trichoderma* sp. F4. The isolated fungi demonstrated potential for synthesizing the hydrolytic enzymes.

## 1. Introduction

Xylan is a noncrystalline complex polysaccharide consisting of a backbone of *β*-D-1, 4-linked xylopyranoside units substituted with acetyl, glucuronosyl, and arabinosyl side chains [1]. Xylans are the main carbohydrate in the hemicellulosic fraction of vegetable tissues and form an interface between lignin and the other polysaccharides. The polysaccharides are mainly found in secondary plant cell walls, and their characteristic of adhesion helps to maintain the integrity of the cellular wall [2]. Cellulose is a linear polymer of D-glucose units linked by 1, 4-*β*-D-glucosidic bond and is crystalline in nature [3]. Cellulose is the main constituent of plants and thus the most abundant biopolymer on earth comprising approximately 35–50% of plant dry weight [4]. Hydrolysis of xylan and cellulose are essential steps towards the efficient utilization of lignocellulosic materials in nature. Lignocellulosic waste forms a large proportion of solid waste in our cities, thus constituting an environmental problem. Studies have shown that conventional waste treatment strategies have failed to ameliorate this problem. The use of microbial enzymes in lignocellulosic waste treatment has been shown to be an alternative that is efficient and cost-effective. Therefore, considering the industrial potentials of xylanases and cellulases, and their potential use in lignocellulolytic waste treatment, it becomes imperative to obtain new enzymes and enzyme-producing microbial strains that produce highly active xylanases and cellulases at low cost. Chemical hydrolysis of lignocellulose is accompanied with the formation of toxic components that are toxic to the environment [5], hence the need to explore the use of microorganisms and their enzymes, which have high specificity, mild reaction conditions, negligible substrate loss, and side product generation and are environmentally friendly [6], in lignocellulose hydrolysis. Xylanases and cellulases are widely abundant in nature; they are produced by bacteria, fungi, protozoa, algae, gastropods, arthropods, nematodes, and so forth [7].

Filamentous fungi have been reported to be good producers of lignocellulolytic enzymes from industrial point of view due to extracellular release of the enzymes, higher yield compared to yeast and bacteria, and also the production of several auxiliary enzymes that are necessary for debranching of substituted polysaccharides [8]. The application of xylanases and cellulases has been mainly considered for the bioconversion of lignocellulosic materials, especially residues and wastes produced by agriculture and forestry to produce higher value products such as ethanol fuel and other chemicals. Other potential applications of the enzymes include bread making, fruit juice extraction, beverage preparation, increasing digestibility of animal feed, converting lignocellulosic substances to feedstock, and fibre separation and in paper and pulp industries.

Therefore, considering the potentials of using xylanases and cellulases in industries and lignocellulolytic waste treatment, it becomes imperative to obtain enzyme-producing microbial strains that produce highly active xylanases and cellulases. Genetic manipulations by classical mutation techniques and by use of recombinant DNA technology have been used to increase the expression levels of a large number of microbial enzymes. The use of modern techniques to improve the production of metabolites does not invalidate the search for wild organisms producing useful metabolites. In fact the screening of naturally occurring microorganisms may be the best way to obtain new strains and/or enzymes for commercial applications [9]. Due to the need to obtain xylanases and cellulases with specific processing characteristics, especially in developing countries with low technological capabilities, this study was undertaken to screen fungal cultures for cellulose and xylan degrading enzymes.

## 2. Materials and Methods

### 2.1. Isolation of Fungi

Fungi were isolated from soil samples, decaying logs of wood, and sawdust using a tenfold serial dilution-plating technique on potato dextrose agar (PDA) plates into which 30 *μ*g of chloramphenicol was added. This was incubated at room temperature, that is, 28 ± 2°C [10]. The culture was observed daily and fungal growth was subcultured onto fresh plates of PDA until pure isolates were obtained. The pure cultures were then transferred to PDA slants and maintained by subculturing every four weeks.

### 2.2. Identification of Fungi

The isolated fungi were identified after growth on PDA medium by observing their macroscopic (colour, texture, appearance, and diameter of colonies) and microscopic (microstructures) characteristics according to Barnett and Hunter [11], Domsch et al. [12], Lieckfeldt et al. [13], and Jaklitsch et al. [14]. Smears of the isolated fungi were prepared in Lactophenol cotton blue and examined with the X40 objectives of a compound binocular microscope for microscopic appearance.

The relative occurrence of the fungi was determined by the following formula:

(1) $$\begin{array}{c} \text{Relative occurrence}\left(\%\right)=\frac{T_{o}}{T_{i}}\times 100, \end{array}$$

where *T*_*o*_ is the total number of occurrence of a particular fungus and *T*_*i*_ is the total number of isolates of all fungi [15].

### 2.3. Basal Medium for Enzyme Production

The basal medium composition for production of enzyme by submerged fermentation was based on a modified previous medium [16] containing (gL^−1^): 1.4 (NH_4_)_2_SO_4_, 2 KH_2_PO_4_, 0.3 CaCl_2_, 0.3 MgS0_4_·7H_2_0, 2 CoCl_2_, and 1 mL trace elements. The composition of the trace element solution was (gL^−1^) MnSO_4_·H_2_O, 1.56; FeSO_4_·7H_2_O, 5; ZnSO_4_·7H_2_O, 1.4. The carbon source, carboxymethyl cellulose (CMC) (Sigma Chemical Co.) for cellulase production, was added to the basal medium at 1% concentration and then sterilized at 121°C for 15 min. 1 mL of sterilized trace elements was then added to the medium after cooling. Oat-spelt xylan (Sigma Chemical Co.) was used as carbon source instead of CMC in the medium for production of xylanase.

### 2.4. Enzyme Production by Submerged Fermentation

Submerged fermentation for enzyme production was performed as previously described [17]. Twenty millilitres (20 mL) of the sterile basal medium prepared as earlier described (with the appropriate carbon source) in a 50 mL conical flask was inoculated with 1 mL of standardized fungal spore suspension (1 × 10^7^ spores mL^−1^).

After statically incubating the conical flasks for 6 days at 30°C the content of each flask was filtered through Whatman filter paper no.1. The supernatant solutions were stored at 4°C for subsequent use as crude enzyme preparations. The experiments were performed in duplicate for all the fungal isolates.

### 2.5. Xylanase Activity Assay

Xylanase activity was determined by incubating 0.1 mL of culture filtrate with 0.9 mL of 1% (w/v) oat-spelt xylan (Sigma Chemical Co., St. Louis, Mo.) in 0.05 M citrate buffer, pH 5.0 at 50°C for 30 min [18]. The reaction was terminated by adding 1 mL of dinitrosalicylic acid (DNSA) reagent. The reaction mixture was then placed in a boiling water bath at 100°C for 5 min and thereafter cooled to room temperature [19]. Absorbance was read at 540 nm using a PYE UNICAM SP6-250 visible spectrophotometer. Xylose (Sigma Chemical Co., St. Louis, Mo.) was used as standard. Xylanase activity was expressed as 1 *μ*mol of reducing sugar (xylose equivalent) released per minute per milliliter of enzyme solution.

### 2.6. Carboxymethyl Cellulase Activity

Carboxymethyl cellulase (CMCase) activity was measured by determining the amount of reducing sugar released from low viscosity carboxymethyl cellulose (CMC) (Sigma chemical Co., St. Louis, Mo.). The reaction mixture consisted of 0.9 mL 1% (w/v) CMC in 0.1 M citrate buffer, pH 5.0, and 0.1 mL culture filtrate [20]. After incubation at 50°C for 30 min the reaction was stopped by addition of 1 mL DNSA acid followed by boiling in a water bath at 100°C for 5 min [19]. After cooling the reaction mixture to room temperature, the absorbance values were read at 540 nm using a PYE UNICAM SP6-250 visible spectrophotometer. Glucose (Sigma Chemical Co., St. Louis, Mo.) was used as standard. One unit (U) of CMCase activity was expressed as 1 *μ*mol of reducing sugar (glucose equivalent) released per minute per milliliter of enzyme solution.

### 2.7. Protein Assay

Protein estimation was carried out by the method of Lowry [21].

## 3. Results and Discussion

### 3.1. Fungal Isolates

A total of 110 fungi were isolated from saw dust, soil, and decaying wood (Table 1). The fungi with the highest relative occurrence are *Trichoderma *sp. (20.0%), *Aspergillus niger* (16.3%), *Trichoderma viride* (13.6%), and *Aspergillus *sp. and *Aspergillus ustus *(10.9%). The relative occurrence of members of the genera *Trichoderma* and *Aspergillus* was 42.6% and 40.8%, respectively, thereby making them the fungi most isolated. The fungi of lowest incidence was *A. fumigatus* (0.9%), followed by *A. flavus, T. pseudokoningii, and Penicillium *sp. (1.8%). The highest occurrence of fungi was recorded from soil samples (52%), followed by saw dust (29%), and least was in decaying wood (19%). Fungi from all six genera observed were isolated from the soil samples. *A. fumigatus, T. pseudokoningii, *and *Penicillium *sp. were isolated from soil samples only, and not from the other samples. *A. flavus, Fusarium *sp., *Rhizopus *sp., and *T. longibrachiatum* were isolated from sawdust and soil samples only. *Aspergillus *sp.,* A. niger, A. ustus, Trichoderma *sp., and *T. viride* were isolated from all the three different types of samples used for the isolation of the fungi. *Mucor* sp. was not isolated from saw dust samples. This finding is in line with previously reported studies [22–26] that members of the genera *Aspergillus and Trichoderma *were the dominant fungi in forest and agricultural soils. Fungi have many different functions in soils, which include either active roles, such as the degradation of dead plant material, or inactive roles where propagules are present in the soil as a resting stage [27].

## 4. Screening of Fungal Isolates for Enzymatic Activities

Cellulolytic and xylanolytic activities of the isolated fungi were determined by estimating the amount of reducing sugar released by the fungi when grown in carboxymethyl cellulose (CMC) and oat-spelt xylan, respectively. Isolates FF2, FF12, and FF6 from decaying logs of wood produced significantly high amount of reducing sugar (1.20 mg mL^−1^, 1.02 mg mL^−1^, and 1.00 mg mL^−1^, resp.). The lowest value of 0.35 mg mL^−1^ reducing sugar from oat-spelt xylan was observed in isolate FF13. A significantly high amount of reducing sugar (0.60 mg mL^−1^) from CMC was given by isolate FF9 and the least (0.10 mg mL^−1^) was given by isolates FF3, FF13, and FF20 (Table 2).

Table 3 shows the amounts of reducing sugar produced during screening of isolated fungi from saw dust for cellulase and xylanase production. Isolates FD26 (1.35 mg mL^−1^) and FD18 (1.30 mg mL^−1^) produced the highest amount of reducing sugar when grown on oat-spelt xylan. Other high reducing sugar producing isolates from oat-spelt xylan were FD7 (1.25 mg mL^−1^), FD12 (1.20 mg mL^−1^), FD16, FD23, and FD25 (1.00 mg mL^−1^). The lowest amount of reducing sugar was produced by isolate FD28 (0.15 mg mL^−1^) acting on oat-spelt xylan. Isolates FD4, FD7, and FD18 produced the highest amount of reducing sugar from CMC. Isolates FD14 (0.75 mg mL^−1^) and FD26 (0.70 mg mL^−1^) were also high reducing sugar producers from CMC. The lowest amount of reducing sugar (0.01 mg mL^−1^) from CMC was produced by isolate FD5.

The amount of reducing sugar produced when screening fungal isolates from soil for hydrolytic enzyme activity is shown in Table 4. The highest amount of reducing sugar was produced by isolate FS3 (1.30 mg mL^−1^) when grown in oat-spelt xylan. Three other high reducing sugar producing isolates from oat-spelt xylan were FS34 and FS48 (1.20 mg mL^−1^), FS7 (1.15 mg mL^−1^), FS10, FS35, and FS50 (1.00 mg mL^−1^). Isolate FS4 produced the least amount of reducing sugar (0.10 mg mL^−1^) from oat-spelt xylan. When the isolated fungi were grown in CMC, isolate FS48 (0.75 mg mL^−1^) was the highest reducing sugar producer, followed by isolates FS47 (0.60 mg mL^−1^) and FS39 (0.55 mg mL^−1^). Isolate FS17 (0.05 mg mL^−1^) was the lowest reducing sugar producer on CMC.

Seventeen [17] fungal isolates that produced high amount of reducing sugar (≥1.00 mg mL^−1^) when grown on oat-spelt xylan were selected for further screening. The protein content of the culture filtrates obtained after growing the selected fungal isolates in basal medium containing oat-spelt xylan was determined and the specific activity of the enzyme produced by the organisms calculated. The result is presented in Table 5. The selected fungal isolate with highest specific activity of 1.30 U mg^−1^ protein was isolate FD18. The xylanase specific activities of isolate FD18 and isolate FD7 (1.22 U mg^−1^) were significantly better (*P* < 0.05) than that of the other isolates. This was followed by isolates FD12 and FS48 with specific activities of 1.08 and 1.05 U mg^−1^ proteins, respectively. The isolates with low specific activities were FF6 (0.54 U mg^−1^ proteins), FD25 (0.63 U mg^−1^ proteins), FS10 (0.65 U mg^−1^ protein), and FF2 (0.68 U mg^−1^ proteins).

The result of the specific activities of seven selected fungal strains that produced high reducing sugar when grown in CMC is shown in Table 6. The isolate FD4 produced a significantly higher (*P* ≤ 0.05) specific activity of 1.23 U mg^−1^ proteins. The lowest specific activity (0.61 U mg^−1^ proteins) was obtained by isolate FD26.

The fungal strains that gave specific activities of ≥1.0 in Tables 5 and 6 were chosen for further studies of their enzymatic potentials. The strains were identified as *Aspergillus ustus *Fs48, *Trichoderma* sp. Fd4, *Trichoderma* sp. Fd7, *Aspergillus ustus *Fd12, and *Trichoderma viride* Fd18.

Aspergilli are known to produce an extensive range of plant cell wall degrading enzymes. Many species of the genus have been identified to possess all component of the cellulase complex [28]. *Trichoderma *has been listed as a common and effective cellulase producer [25, 29–32]. There are many reports on isolation of cellulose and xylanase producing fungi from soil, lignocellulosic waste from the vinegar industry, waste paper, cotton waste, bagasse, and leaf litters [32]. Fungi are well known agents of decomposition of particularly xylan and cellulose containing organic matter. The decomposition of xylan and cellulose is of significance in the biological carbon cycle. Xylan and cellulose degrading enzymes have been used in food processing, detergent formulation, textile production, feed preparation, production of wine, beer, and fruit juice, and in bioconversion of lignocelluloses to fuel ethanol [31, 33].

In this study, *Trichoderma *and *Aspergillus *had a higher relative rate of occurrence in saw dust, log of wood, and soil. The organisms also produced xylanase and cellulase with high specific activities compared to the other isolates. The fungal cultures will be further studied for their enzymatic potentials in the bioconversion of lignocellulosic waste to useful products.

## Figures and Tables

**Table 1:** Relative occurrence (%) of fungi isolated from soil, saw dust, and decaying wood.

Probable identity	Source/occurrence			
	Saw dust	Soil	Decaying wood	Total
*Aspergillus *sp.	2 (1.8%)*	7 (6.4%)	3 (2.7%)	12 (10.9%)
*Aspergillus flavus *	1 (0.9%)	1 (0.9%)	—	2 (1.8%)
*Aspergillus fumigatus *	—	1 (0.9%)	—	1 (0.9%)
*Aspergillus niger *	7 (6.4%)	8 (7.3%)	3 (2.7%)	18 (16.3%)
*Aspergillus ustus *	6 (5.5%)	4 (3.6%)	2 (1.8%)	12 (10.9%)
*Rhizopus *sp.	1 (0.9%)	3 (2.7%)	—	4 (3.6%)
*Trichoderma *sp.	7 (6.4%)	9 (8.2%)	6 (5.5%)	22 (20.0%)
*Trichoderma harzianum *	1 (0.9%)	2 (1.8%)	1 (0.9%)	4 (3.6%)
*Trichoderma longibrachiatum *	1 (0.9%)	3 (2.7%)	—	4 (3.6%)
*Trichoderma pseudokoningii *	—	2 (1.8%)	—	2 (1.8%)
*Trichoderma viride *	5 (4.5%)	7 (6.4%)	3 (2.7%)	15 (13.6%)
*Mucor *sp.	—	6 (5.5%)	3 (2.7%)	9 (8%)
*Fusarium *sp.	1 (0.9%)	2 (1.8%)	—	3 (2.7%)
*Penicillium *sp.	—	2 (1.8%)	—	2 (1.8%)

Total	32 (29.0%)	57 (52%)	21 (19.0%)	110 (100%)

*Figure in parenthesis represent the relative occurrence of the fungi in percentages.

**Table 2:** Reducing sugar (mg/mL) produced during screening of fungi isolated from decaying logs of wood for cellulase and xylanase production.

Fungal isolate	Substrate/reducing sugar (mg/mL)	
	Oat-spelt xylan	CMC
FF1	0.55 ± 0.00^hi^	0.20 ± 0.00^gh^
FF2	1.20 ± 0.14^a^	0.35 ± 0.07^de^
FF3	0.50 ± 0.07^i^	0.10 ± 0.00^i^
FF4	0.90 ± 0.00^bc^	0.30 ± 0.00^ef^
FF5	0.65 ± 0.00^fgh^	0.28 ± 0.01^f^
FF6	1.00 ± 0.14^b^	0.15 ± 0.07^hi^
FF7	0.90 ± 0.07^bc^	0.35 ± 0.00^de^
FF8	0.85 ± 0.00^cd^	0.30 ± 0.00^ef^
FF9	0.90 ± 0.00^bc^	0.60 ± 0.03^a^
FF10	0.80 ± 0.07^cde^	0.40 ± 0.04^cd^
FF11	0.85 ± 0.04^cd^	0.45 ± 0.07^bc^
FF12	1.02 ± 0.03^b^	0.40 ± 0.00^cd^
FF13	0.35 ± 0.00^j^	0.10 ± 0.00^i^
FF14	0.75 ± 0.00^def^	0.25 ± 0.00^fg^
FF15	0.60 ± 0.00^ghi^	0.15 ± 0.00^hi^
FF16	0.90 ± 0.03^bc^	0.50 ± 0.03^b^
FF17	0.60 ± 0.01^ghi^	0.15 ± 0.03^hi^
FF18	0.75 ± 0.03^*def*^	0.35 ± 0.00^de^
FF19	0.65 ± 0.00^fgh^	0.40 ± 0.01^cd^
FF20	0.70 ± 0.01^efg^	0.10 ± 0.00^i^
FF21	0.85 ± 0.00^cd^	0.45 ± 0.00^bc^

^∗^Each value is a mean of two replicates; ± stands for standard deviation among replicates; means followed by different letters within each column differ significantly at *P* ≤ 0.05; CMC: carboxymethyl cellulose.

**Table 3:** Reducing sugar (mg/mL) produced during screening of fungi isolated from saw dust for cellulase and xylanase production.

Fungal isolate	Substrate/reducing sugar (mg/mL)	
	Oat-spelt xylan	CMC
FD1	*0.75 ± 0.00^gh^	0.35 ± 0.01^gh^
FD2	0.90 ± 0.00^e^	0.40 ± 0.01^fg^
FD3	0.80 ± 0.03^fg^	0.40 ± 0.00^fg^
FD4	0.90 ± 0.00^e^	0.85 ± 0.00^a^
FD5	0.33 ± 0.02^lm^	0.01 ± 0.01^n^
FD6	0.90 ± 0.01^e^	0.50 ± 0.01^de^
FD7	1.25 ± 0.00^bc^	0.85 ± 0.07^a^
FD8	0.65 ± 0.00^ij^	0.05 ± 0.01^mn^
FD9	0.65 ± 0.00^ij^	0.25 ± 0.00^ij^
FD10	0.85 ± 0.17^gh^	0.15 ± 0.00^kl^
FD11	0.80 ± 0.00^fg^	0.60 ± 0.03^c^
FD12	1.20 ± 0.03^c^	0.55 ± 0.01^cd^
FD13	0.80 ± 0.00^fg^	0.40 ± 0.01^fg^
FD14	0.70 ± 0.00^hi^	0.75 ± 0.03^b^
FD15	0.45 ± 0.07^k^	0.20 ± 0.03^jk^
FD16	1.00 ± 0.00^d^	0.35 ± 0.03^gh^
FD17	0.75 ± 0.00^gh^	0.35 ± 0.00^gh^
FD18	1.30 ± 0.00^ab^	0.85 ± 0.07^a^
FD19	0.60 ± 0.00^j^	0.35 ± 0.00^gh^
FD20	0.50 ± 0.01^k^	0.10 ± 0.00^lm^
FD21	0.75 ± 0.06^gh^	0.30 ± 0.01^hi^
FD22	0.65 ± 0.65^ij^	0.30 ± 0.00^hi^
FD23	1.00 ± 0.00^d^	0.10 ± 0.00^lm^
FD24	0.70 ± 0.00^hi^	0.05 ± 0.00^mn^
FD25	1.00 ± 0.00^d^	0.10 ± 0.00^lm^
FD26	1.35 ± 0.00^a^	0.70 ± 0.00^b^
FD27	0.85 ± 0.03^ef^	0.45 ± 0.00^ef^
FD28	0.15 ± 0.00^n^	0.10 ± 0.00^lm^
FD29	0.25 ± 0.00^m^	0.10 ± 0.00^lm^
FD30	0.35 ± 0.01^l^	0.15 ± 0.07^kl^
FD31	0.75 ± 0.01^gh^	0.30 ± 0.04^hi^
FD32	0.85 ± 0.07^ef^	0.28 ± 0.04^i^

*Each value is a mean of two replicates; ± stands for standard deviation among replicates; means followed by different letters within each column differ significantly at *P* ≤ 0.05; CMC: carboxymethyl cellulose.

**Table 4:** Reducing sugar (mg/mL) produced during screening of fungi isolated from soil for cellulase and xylanase production.

Fungal isolate	Substrate/reducing sugar (mg/mL)	
	Oat-spelt xylan	CMC
FS1	*0.30 ± 0.00^k^	0.05 ± 0.00^k^
FS2	0.70 ± 0.00^gh^	0.20 ± 0.00^hi^
FS3	1.30 ± 0.07^a^	0.35 ± 0.03^ef^
FS4	0.10 ± 0.00^m^	0.15 ± 0.04^ij^
FS5	0.90 ± 0.03^cde^	0.30 ± 0.01^fg^
FS6	0.94 ± 0.04^cd^	0.35 ± 0.01^ef^
FS7	1.15 ± 0.21^b^	0.45 ± 0.00^cd^
FS8	0.80 ± 0.00^efg^	0.25 ± 0.01^gh^
FS9	0.70 ± 0.00^gh^	0.40 ± 0.00^de^
FS10	1.00 ± 0.00^c^	0.45 ± 0.00^cd^
FS11	0.85 ± 0.07^*def*^	0.30 ± 0.04^fg^
FS12	0.60 ± 0.00^hi^	0.20 ± 0.00^hi^
FS13	0.85 ± 0.06^*def*^	0.35 ± 0.03^ef^
FS14	0.55 ± 0.00^i^	0.15 ± 0.00^ij^
FS15	0.40 ± 0.00^j^	0.20 ± 0.07^hi^
FS16	0.20 ± 0.00^l^	0.15 ± 0.03^ij^
FS17	0.10 ± 0.03^m^	0.05 ± 0.00^k^
FS18	0.50 ± 0.00^ij^	0.10 ± 0.00^jk^
FS19	0.50 ± 0.00^ij^	0.10 ± 0.01^jk^
FS20	0.95 ± 0.07^cd^	0.30 ± 0.00^fg^
FS21	0.95 ± 0.00^cd^	0.20 ± 0.03^hi^
FS22	0.90 ± 0.00^cde^	0.45 ± 0.07^cd^
FS23	0.55 ± 0.03^i^	0.15 ± 0.03^ij^
FS24	0.70 ± 0.00^gh^	0.20 ± 0.00^hi^
FS25	0.80 ± 0.01^efg^	0.35 ± 0.00^ef^
FS26	0.50 ± 0.00^ij^	0.35 ± 0.04^ef^
FS27	0.80 ± 0.00^efg^	0.35 ± 0.03^ef^
FS28	0.60 ± 0.00^hi^	0.20 ± 0.00^hi^
FS29	0.70 ± 0.03^gh^	0.25 ± 0.00^gh^
FS30	0.80 ± 0.01^efg^	0.40 ± 0.03^de^
FS31	0.60 ± 0.00^hi^	0.30 ± 0.03^fg^
FS32	0.75 ± 0.00^fg^	0.65 ± 0.07^a^
FS33	0.70 ± 0.01^gh^	0.30 ± 0.03^fg^
FS34	1.20 ± 0.14^b^	0.55 ± 0.00^b^
FS35	1.00 ± 0.00^c^	0.10 ± 0.00^jk^
FS36	0.40 ± 0.00^j^	0.35 ± 0.00^ef^
FS37	0.70 ± 0.00^gh^	0.35 ± 0.00^ef^
FS38	0.50 ± 0.03^ij^	0.10 ± 0.00^jk^
FS39	0.90 ± 0.00^cde^	0.55 ± 0.03^b^
FS40	0.75 ± 0.00^fg^	0.25 ± 0.00^gh^
FS41	0.70 ± 0.00^gh^	0.50 ± 0.01^bc^
FS42	0.60 ± 0.00^hi^	0.40 ± 0.03^de^
FS43	0.50 ± 0.03^ij^	0.20 ± 0.03^hi^
FS44	0.70 ± 0.00^gh^	0.10 ± 0.00^jk^
FS45	0.35 ± 0.00^l^	0.40 ± 0.00^fg^
FS46	0.75 ± 0.00^gh^	0.10 ± 0.00^lm^
FS47	0.75 ± 0.03^c^	0.60 ± 0.00^bc^
FS48	1.20 ± 0.00^a^	0.75 ± 0.00^a^
FS49	0.60 ± 0.00^de^	0.50 ± 0.00^bc^
FS50	1.00 ± 0.01^b^	0.30 ± 0.00^fg^
FS51	0.50 ± 0.07^f^	0.55 ± 0.14^b^
FS52	0.65 ± 0.00^d^	0.15 ± 0.07^ij^
FS53	0.50 ± 0.00^f^	0.50 ± 0.00^bc^
FS54	0.55 ± 0.03^ef^	0.30 ± 0.14^fg^
FS55	0.20 ± 0.00^h^	0.50 ± 0.00^bc^
FS56	0.65 ± 0.03^d^	0.10 ± 0.00^lm^
FB57	0.35 ± 0.00^g^	0.30 ± 0.00^c^

*Each value is a mean of two replicates; ± stands for standard deviation among replicates; means followed by different letters within each column differ significantly at *P* ≤ 0.05; CMC: carboxymethyl cellulose.

**Table 5:** Specific activities of xylanase produced by fungal isolates.

Fungal isolates	Xylanase (U mL^−1^)	Protein (mg mL^−1^)	Specific activity (U mg^−1^ protein)
FS3	*338 ± 2.83^ab^	380 ± 0.00^dc^	0.89 ± 0.00^*def*^
FS7	300 ± 8.28^dc^	310 ± 4.14^de^	0.97 ± 0.01^cd^
FS10	260 ± 0.00^e^	400 ± 13.14	0.65 ± 0.03^h^
FS34	312 ± 6.97^bcd^	420 ± 8.28^abc^	0.74 ± 0.00^g^
FS35	260 ± 2.43^de^	288 ± 1.31^e^	0.90 ± 0.01^def^
FD7	330 ± 7.07^abc^	270 ± 7.07^e^	1.22 ± 0.01^a^
FD12	312 ± 6.97^bcd^	288 ± 8.28^e^	1.08 ± 0.03^b^
FD16	260 ± 4.14^e^	305 ± 1.21^de^	0.85 ± 0.03^ef^
FD18	338 ± 1.31^ab^	261 ± 4.14^e^	1.30 ± 0.03^a^
FD23	260 ± 4.14^e^	282 ± 5.66^e^	0.94 ± 0.03^de^
FD25	260 ± 7.07^e^	410 ± 0.00^abc^	0.63 ± 0.01^h^
FD26	352 ± 4.14^a^	400 ± 7.07	0.88 ± 0.11^*def*^
FF2	312 ± 0.00^bcd^	460 ± 1.21^ab^	0.68 ± 0.03^gh^
FF6	260 ± 2.83^e^	480 ± 9.90^a^	0.54 ± 0.03^i^
FF12	265.2 ± 0.00^e^	395 ± 7.07^bc^	0.67 ± 0.03^gh^
FS48	318 ± 8.49^abcd^	298 ± 2.83^e^	1.05 ± 0.07^bc^
FS50	260 ± 0.00^e^	310 ± 2.83^de^	0.84 ± 0.03^f^

*Each value is a mean of two replicates; ± stands for standard deviation among replicates; means followed by different letters within each column differ significantly at *P* ≤ 0.05.

**Table 6:** Specific activities of cellulase produced by fungal isolates.

Fungal isolates	Cellulase (U mL^−1^)	Protein (mg mL^−1^)	Specific activity (U mg^−1^ protein)
FS48	*195 ± 2.83^b^	298 ± 2.83^a^	0.65 ± 0.01^cd^
FD4	221 ± 0.00^a^	180 ± 2.83^d^	1.23 ± 0.01^a^
FD7	221 ± 2.83^a^	260 ± 2.83^b^	0.85 ± 0.04^b^
FD11	156 ± 8.49^c^	210 ± 2.83^c^	0.74 ± 0.01^c^
FD14	195 ± 7.07^b^	270 ± 2.83^b^	0.72 ± 0.03^c^
FD18	221 ± 8.49^b^	261 ± 8.49^b^	0.85 ± 0.07^b^
FD26	182 ± 2.83^b^	298 ± 3.83^a^	0.61 ± 0.01^d^

*Each value is a mean of two replicates; ± stands for standard deviation among replicates; means followed by different letters within each column differ significantly at *P* ≤ 0.05.

## References

[1] la Grange DC, Claeyssens M, Pretorius IS, van Zyl WH (2001). Degradation of Xylan to D-Xylose by recombinant *Saccharomyces cerevisiae* coexpressing the *Aspergillus nigerβ*-Xylosidase (*xln*D) and the *Trichoderma reesei* Xylanase II (*xyn*2) genes. *Applied and Environmental Microbiology*.

[2] Goulart AJ, Carmona EC, Monti R (2005). Partial purification and properties of cellulase-free alkaline xylanase produced by *Rhizopus stolonifer* in solid-state fermentation. *Brazilian Archives of Biology and Technology*.

[3] Leschine SB (1995). Cellulose degradation in anaerobic environments. *Annual Review of Microbiology*.

[4] Lynd LR, Weimer PJ, van Zyl WH, Pretorius IS (2002). Microbial cellulose utilization: fundamentals and biotechnology. *Microbiology and Molecular Biology Reviews*.

[5] Ninawe S, Kuhad BC (2005). Use of xylan-rich cost effective agro-residues in the production of xylanase by *Streptomyces cyaneus* SN32. *Journal of Applied Microbiology*.

[6] Kulkarni N, Shendye A, Rao M (1999). Molecular and biotechnological aspects of xylanases. *FEMS Microbiology Reviews*.

[7] Nadia HAE-N, El Sayed MM, Wafaa GS, Gehad HES (2010). Purification and partial characterization of extracellular cellulase free xylanase from *Streptomyces rochei*. *Journal of Applied Sciences Research*.

[8] Haltrich D, Nidetzky B, Kulbe KD, Steiner W, Župančič S (1996). Production of fungal xylanases. *Bioresource Technology*.

[9] Narasimha G, Sridevi A, Buddolla V, Subhosh Chandra M, Rajasekhar Reddy B (2006). Nutrient effects on production of cellulolytic enzymes by *Aspergillus niger*. *African Journal of Biotechnology*.

[10] Johnson LF, Curl EA (1972). *Methods for Research on the Ecology of Soil-Borne Plant Pathogens*.

[11] Barnett JL, Hunter BB (1972). *Illustrated Genera of Imperfect Fungi*.

[12] Domsch KH, Gams W, Anderson T-H (1980). *Compendium of Soil Fungi*.

[13] Lieckfeldt E, Samuels GJ, Nirenberg HI, Petrini O (1999). A morphological and molecular perspective of *Trichoderma viride*: is it one or two species?. *Applied and Environmental Microbiology*.

[14] Jaklitsch WM, Samuels GJ, Dodd SL, Lu B-S, Druzhinina IS (2006). *Hypocrea rufa*/*Trichoderma viride*: a reassessment, and description of five closely related species with and without warted conidia. *Studies in Mycology*.

[15] Das MKL, Prasad JS, Ahmad SK (1997). Endoglucanase production by paper-degrading mycoflora. *Letters in Applied Microbiology*.

[16] Mandels M, Weber J (1969). The production of cellulases. *Advances in Chemistry Series*.

[17] Fernandes Franco P, Malheiros Ferreira H, Ferreira Filho EX (2004). Production and characterization of hemicellulase activities from *Trichoderma harzianum* strain T4. *Biotechnology and Applied Biochemistry*.

[18] Bailey MJ, Biely P, Poutanen K (1992). Interlaboratory testing of methods for assay of xylanase activity. *Journal of Biotechnology*.

[19] Miller GL (1959). Use of dinitrosalicylic acid reagent for determination of reducing sugar. *Analytical Chemistry*.

[20] Ghose TK (1987). Measurement of cellulase activities. *Pure and Applied Chemistry*.

[21] Lowry OH, Rosebrough NJ, Farr AL, Randall RJ (1951). Protein measurement with the Folin phenol reagent. *The Journal of Biological Chemistry*.

[22] Qiao H, Tian C, Luo Y, Sun J, Feng X (2008). Diversity of soil microorganisms in natural *Populus euphratica* forests in Xinjiang, northwestern China. *Frontiers of Forestry in China*.

[23] Jaime-Garcia R, Cotty PJ (2010). Crop rotation and soil temperature influence the community structure of *Aspergillus flavus* in soil. *Soil Biology and Biochemistry*.

[24] Kausar H, Sariah M, Mohd Saud H, Zahangir Alam M, Razi Ismail M (2010). Development of compatible lignocellulolytic fungal consortium for rapid composting of rice straw. *International Biodeterioration and Biodegradation*.

[25] Reanprayoon P, Pathomsiriwong W (2012). Tropical soil fungi producing cellulase and related enzymes in biodegradation. *Journal of Applied Sciences*.

[26] Gautam SP, Bundela PS, Pandey AK, Jamaluddin J, Awasthi MK, Sarsaiya S (2012). Diversity of cellulolytic microbes and the biodegradation of municipal solid waste by a potential strain. *International Journal of Microbiology*.

[27] Vega K, Villena GK, Sarmiento VH, Ludena Y, Vera N, Gutierrez-Correa M (2012). Production of alkaline cellulase by fungi isolated from an undisturbed rain forest of peru. *Biotechnology Reseach Journal*.

[28] De Vries RP, Visser J (2001). *Aspergillus* enzymes involved in degradation of plant cell wall polysaccharides. *Microbiology and Molecular Biology Reviews*.

[29] Yalpani M (1987). Development and prospect in enzymatic biopolymer modification. *Industrial Polysaccharide*.

[30] Nwodo-Chinedu S, Okochi VI, Smith HA, Omidiji O (2005). Ioslation of cellulolytic microfungi involved in wood waste decomposition: prospects for enzymatic hydrolysis of cellulosic waste. *International Journal of Biomedical and Health Sciences*.

[31] Sri Laksmi A, Narasimha G (2012). Production of cellulases by fungal cultures isolated from forest litter. *Annals of Forest Research*.

[32] Chandel K, Jandaik S, Kumari V (2013). Isolation, purification and screening of cellulolytic fungi from mushroom compost for production of enzyme (cellulose). *International Journal of Current Research*.

[33] Khokhar I, Haider MS, Mushtaq S, Mukhtar I (2012). Isolation and screening of highly cellulolytic filamentous fungi. *Scholarly Journal of Agricultural Science*.

